# Neurotrophins in Neurodevelopmental Disorders: A Narrative Review of the Literature

**DOI:** 10.3390/ijms26178335

**Published:** 2025-08-28

**Authors:** Fabiola Panvino, Roberto Paparella, Francesca Tarani, Chiara Lombardi, Giampiero Ferraguti, Francesco Pisani, Marco Fiore, Rouzha Pancheva, Ignazio Ardizzone, Luigi Tarani

**Affiliations:** 1 Department of Human Neuroscience, Sapienza University of Rome, 00185 Roma, Italy; fabiola.panvino@uniroma1.it (F.P.);; 2 Department of Maternal Infantile and Urological Sciences, Sapienza University of Rome, 00185 Roma, Italy; roberto.paparella@uniroma1.it (R.P.);; 3 Department of Experimental Medicine, Sapienza University of Rome, 00185 Roma, Italy; 4 Institute of Biochemistry and Cell Biology (IBBC-CNR), c/o Department of Sensory Organs, Sapienza University of Rome, 00185 Roma, Italy; 5 Department of Hygiene and Epidemiology, Faculty of Public Health, Medical University Prof Dr Paraskev Stoyanov, 9002 Varna, Bulgaria

**Keywords:** neurotrophins, brain-derived neurotrophic factor, nerve growth factor, neurodevelopmental disorders, attention-deficit/hyperactivity disorder, autism spectrum disorder, intellectual disability, tic disorders

## Abstract

Neurodevelopmental disorders (NDDs), including attention-deficit/hyperactivity disorder (ADHD), autism spectrum disorder (ASD), intellectual disability (ID), and tic disorders, comprise a range of conditions that originate in early childhood and impact cognitive, behavioral, and social functioning. Despite their clinical heterogeneity, they often share common molecular and neurobiological framework. This narrative review aims to examine the role of neurotrophins—particularly the brain-derived neurotrophic factor, nerve growth factor, and related molecules—in the pathophysiology of NDDs, and to explore their potential as biomarkers and therapeutic targets. A comprehensive literature search was conducted using PubMed, Scopus, and Web of Science, including both clinical and preclinical studies. Neurotrophins are critically involved in brain development, influencing neurogenesis, synaptic plasticity, and neuronal survival. Dysregulation in their signaling pathways has been associated with core features of ASD and ADHD and may modulate cognitive outcomes in ID. Emerging evidence also supports a role for neuroimmune interactions and neurotrophic dysfunction in tic disorders. However, findings across studies remain inconsistent due to methodological variability and limited longitudinal data. Future research should aim for standardized methodologies and stratified, longitudinal designs to clarify their role across developmental stages and clinical phenotypes.

## 1. Introduction

Neurodevelopmental disorders (NDDs) comprise a wide spectrum of neurological and psychiatric conditions which influence brain development, leading to delays or impairments in cognitive, social, and motor functioning [1,2]. NDDs typically manifest during early childhood and can persist throughout life with a steady course [1,3].

Currently, the Diagnostic and Statistical Manual of Mental Disorders, Fifth Edition, Text Revision (DSM-5-TR) [4] includes attention-deficit/hyperactivity disorder (ADHD), autism spectrum disorder (ASD), intellectual disability (ID) and tic disorders.

Despite the heterogeneity of NDDs in terms of epidemiology, clinical features, causes, treatment responses, and outcomes, there is a significant overlap in symptoms, likely due to shared multifactorial origins, with a complex interplay of genetic, neurobiological, and environmental factors [5,6,7].

Neurobiological processes involved in the neurodevelopmental trajectory begin early in life, even before birth and include neuronal processes such as neurogenesis, neuronal survival, synaptogenesis, myelination, and activity-dependent forms of synaptic plasticity, which are regulated by numerous biological factors. Among them, neurotrophic factors (polypeptide growth factors) play a crucial role, influencing cognitive, behavioral, and emotional maturation in children. The dysregulation of neurotrophic signaling pathways has been implicated in the pathophysiology of NDDs [8,9,10,11]. This narrative review studies the role of neurotrophins in neurodevelopment, focusing on their potential as biomarkers and the emerging therapeutic approaches. Furthermore, we investigate the connection between neurotrophins and the NDDs mentioned earlier, offering an in-depth analysis of their implications in these conditions.

## 2. Materials and Methods

For this narrative review, we conducted a comprehensive literature search using MEDLINE/PubMed, Scopus, and Web of Science to identify studies exploring the role of neurotrophic factors in neurodevelopmental disorders (NDDs), with a specific focus on pediatric populations. The search was performed until August 2025, without restrictions on publication year, to ensure the inclusion of both foundational and recent studies.

We used a combination of Medical Subject Headings (MeSH) and free-text terms. The primary MeSH terms included “receptors, nerve growth factor (D017475)”, “nerve growth factor (D020932)”, “brain-derived neurotrophic factor (D019208)”, “neurodevelopmental disorders (D065886)”, “attention deficit disorder with hyperactivity (D001289)”, “autism spectrum disorder (D001321)”, “intellectual disability (D008607)”, “tic disorders (D013981)”, and “pediatrics (D010372)”. These were combined using Boolean operators (AND, OR) to optimize sensitivity and specificity.

Eligible studies included original research articles (randomized and non-randomized clinical trials, prospective and retrospective cohort studies, case–control studies, and cross-sectional studies) that investigated neurotrophins in the context of NDDs. Due to the relative scarcity of clinical studies, we also considered relevant in vitro and preclinical studies when they offered mechanistic insights applicable to pediatric neurodevelopment.

A narrative review was preferred over a systematic review approach due to the significant heterogeneity across available studies in terms of population age ranges, methodological designs, and biomarker measurement techniques. Moreover, many areas in the field remain underexplored—particularly the lack of clinical studies—and a narrative synthesis allows for the integration of clinical, preclinical, and mechanistic evidence relevant to pediatric neurodevelopment.

Exclusion criteria were non-English language manuscripts; studies not providing specific data on neurotrophic factors; and non-peer-reviewed sources such as conference abstracts, books, letters to the editor, and editorials.

The selection process was conducted independently by two authors (R.P. and F.Pa.) in two stages: (1) the screening of titles and abstracts and (2) full-text reviews of potentially eligible articles. Disagreements were resolved by discussion or consultation with a third author (C.L.). A total of 196 articles were included in the final review, encompassing molecular mechanisms, clinical correlations, and emerging therapeutic strategies involving neurotrophic pathways in pediatric NDDs. To avoid confusion between species, throughout the review we explicitly indicated when evidence was derived from animal or in vitro studies rather than human clinical data, and we refrained from extrapolating animal findings to human cognition unless clearly stated.

## 3. Neurotrophins: Biology and Function

Neurotrophins are a family of growth factors that includes the nerve growth factor (NGF), brain-derived neurotrophic factor (BDNF), neurotrophin-3 (NT-3), NT-4/5, and glial-derived neurotrophic factor (GDNF). These molecules are secreted by both neuronal and non-neuronal cells (e.g., astrocytes, oligodendrocytes, and endothelial cells) [8,12,13] and are initially produced as precursor proteins, known as proneurotrophins. Neurotrophins play a key role in the differentiation, growth, and survival of these cells from the early stages of pregnancy [14,15]. Several studies have demonstrated that neurotrophins are produced by the mother, supporting the preimplantation stage by regulating implantation, maternal immunity, and modulating vascular growth [14,16], as well as promoting early embryonic development by regulating angiogenesis and vessel stabilization [17,18,19,20]. Neurotrophin production is further supported by the placenta [21]. The differentiation of neural progenitor cells in the third week of gestation marks the beginning of brain development in the fetus [22] and is strictly regulated by BDNF and NGF in both the peripheral and central nervous systems [23,24,25].

Proneurotrophins and mature neurotrophins bind to two classes of receptors: the p75 neurotrophin receptor (p75NTR) and Tyrosine receptor kinases (Trk receptors) [26]. This binding activates to two distinct mechanisms, known as the genomic and non-genomic pathways [27]. The genomic pathway is a slower mechanism that regulates cell growth, survival, and signaling, whereas the non-genomic pathway is involved in neuromodulation.

Notably, the expression of p75NTR begins during pregnancy, from the start of the third month of gestation, and is exhibited by a group of early-born glutamatergic neurons in the subplate zone beneath the cortical plate [28]. It regulates the extension and orientation of subcortical axons through axon-repulsive agents (e.g., myelin-associated glycoprotein) [28]. During central nervous system (CNS) development, p75NTR is widely expressed in both neurons and glial cells. In adulthood, it is expressed in neurons and astrocytes of the hippocampus, as well as in cholinergic neurons of the basal forebrain, which are connected to several cortical areas involved in the regulation of working memory, visual discrimination, and attention [29,30,31,32]. Additionally, p75NTR regulates the survival of sensory neurons and the development of the eye [30].

**Figure 1:**
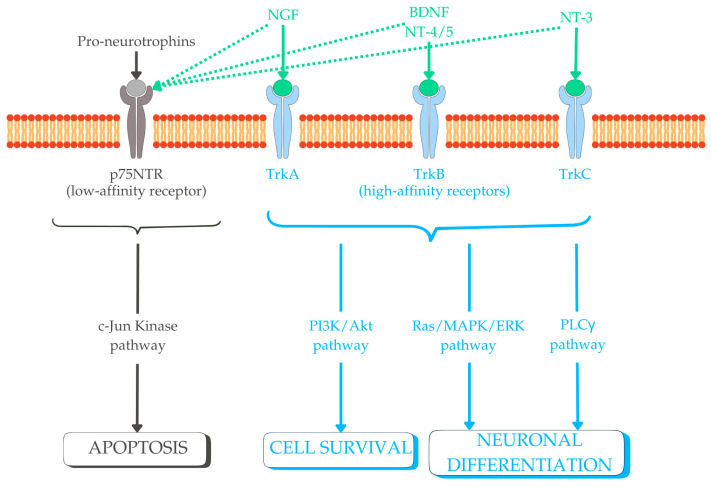
Schematic representation of the main classical neurotrophins, their receptors, and downstream signaling pathways involved in neurodevelopment (glial cell line-derived neurotrophic factor (GDNF) not reported, as it belongs to the GDNF family ligands and signals via other receptors). Abbreviations: BDNF, brain-derived neurotrophic factor; NGF, nerve growth factor; NT-3, neurotrophin-3; NT-4/5, neurotrophin-4/5; PI3K/Akt, phosphoinositide 3-kinase/protein kinase B; PLCγ, phospholipase C gamma; Ras/MAPK/ERK, rat sarcoma/mitogen-activated protein kinase/extracellular signal-regulated kinase.

Proneurotrophins interact with p75NTR with a high affinity, initiating signaling through p53, caspase, and c-Jun N-terminal kinase pathways [33], leading to apoptosis and negatively regulating neuronal growth and synaptic plasticity. On the other hand, mature neurotrophins bind with p75NTR with a low affinity [27,34,35,36,37,38,39,40]. This interaction, however, is essential for activating Trk receptors, leading to phosphorylation and the pairing of intracellular tyrosine residues, triggering downstream signaling cascades (e.g., Ras/MAPK/ERK, PI3K/Akt, and PLCγ pathways) responsible for cell differentiation, survival, and growth. Each neurotrophin binds with its specific Trk receptor: NGF has a high affinity for TrkA, BDNF for TrkB, NT-3 for TrkC, and NT-4 for TrkB. Trk receptors are expressed in the hippocampus, cerebellum, spinal cord, and dorsal root ganglia (DRG) during both CNS and peripheral nervous system (PNS) development. Their distribution and intensity start from the first trimester of gestation and evolve progressively with CNS and PNS maturation [19,41] (Figure 1).

The non-genomic pathway is rapidly activated by the binding of high-affinity Trk receptors, modulating neuronal function through plasma membrane receptors (e.g., NMDA receptors) and cationic channels (e.g., Ca^2+^ influx and voltage-gated Na^+^ channels) [27].

## 4. Neurotrophins During Pregnancy and Early Perinatal Life

Since the earliest stages of implantation, multiple growth factors interact with cytokines, steroid hormones, and adhesion molecules to coordinate endometrial receptivity and trophoblast invasion [42,43,44]. This complex signaling cascade is essential for successful embryo attachment, maturation of the feto-placental unit, and fetal growth [14].

Among growth factors, neurotrophins are expressed not only in embryonic tissues but also at maternal–fetal interfaces, including the decidua and placenta [24,45,46,47]. They contribute to implantation, modulation of maternal immunity, and vascular development. In particular, NGF, BDNF, and NT-3 support placental development and maturation by regulating angiogenesis and vessel stabilization, either directly or via vascular endothelial growth factor (VEGF)-dependent pathways [16,18,20,48]. Neurotrophins are also considered “angioneurins” due to their dual role in promoting both vascular and neuronal development [49]. From the first trimester, they participate in the survival of neural progenitors and the growth of axons [17,19,22]. Higher BDNF levels have been detected in the third trimester and in neonates whose mothers received a complete course of antenatal corticosteroids; conversely, low BDNF levels have been associated with chorioamnionitis and mild intraventricular hemorrhage (IVH) [50]. NT-3 levels also appear to be higher in term infants [51].

Maternal factors such as infection, obstetric conditions, and metabolic status can modulate fetal neurotrophin levels. In animal models, maternal infection alters BDNF and NGF expression in the fetal brain and maternal–fetal unit, potentially affecting synaptic development and increasing the risk of neurodevelopmental disorders [52]. In humans, umbilical cord BDNF levels are influenced by gestational age and mode of delivery, suggesting a role in the neuroendocrine cascade of parturition [46]. Altered neurotrophin levels have also been reported in preeclampsia, possibly through interactions with neuropeptides such as angiotensin II and neuropeptide Y, contributing to impaired vascular function [53,54].

Maternal nutrition further influences fetal neurotrophin expression. Diets rich in omega-3 fatty acids and vitamin B12 increase NGF and BDNF levels and support brain development [55,56,57,58,59,60], whereas high-fat or folate-imbalanced diets reduce neurotrophin expression and impair neurogenesis and plasticity [61,62].

## 5. Neurotrophins Across Postnatal Development

After birth, neurotrophins regulate synaptic plasticity, neurotransmitter regulation, and learning-related processes [63,64,65,66].

BDNF enhances the survival and growth of serotonergic [67,68], dopaminergic [69,70], and cholinergic neurons [71], and promotes dendritic spine formation, which is crucial for memory and cognitive flexibility [72,73,74]. Impairments in BDNF signaling are associated with disrupted synaptic structure, memory deficits, and an increased risk for neurodevelopmental disorders [75,76,77].

In the developing hippocampus, BDNF is essential for the regulation of neurogenesis, particularly in the dentate gyrus. Mice with reduced BDNF expression exhibit lower neurogenic activity [78], while BDNF or NGF infusion enhances neurogenesis and neuronal differentiation [79,80]. NT-3, predominantly expressed in the dentate gyrus, contributes specifically to the differentiation of new neurons [81]. A summary of the main neurotrophins, their primary receptors, biological functions, and developmental roles is provided in Table 1.

Neurotrophin expression peaks in early life and progressively declines throughout development and aging. For example, in humans, BDNF and TrkB mRNA levels in the temporal cortex are highest in neonates and gradually decrease with age [90]. Similarly, proteomic analyses in mice reveal a reduction in hippocampal and cortical BDNF in mid-life [91]. These early-life peaks in neurotrophin availability may be critical for shaping the foundational architecture of the brain. In humans, the hippocampus is the principal neurogenic region implicated in spatial memory-related plasticity across development. By contrast, the subventricular zone (SVZ) is a prominent neurogenic niche in rodents—supporting, for example, olfactory bulb neurogenesis [92]—but SVZ neurogenesis in humans appears to decline rapidly after infancy, and its contribution to memory remains uncertain.

## 6. Neurotrophins in ADHD

ADHD affects approximately 5–7% of children worldwide and usually persists into adulthood in nearly two-thirds of cases [93]. It is characterized by persistent patterns of inattention, hyperactivity, and impulsiveness that interfere with functioning or development. These core symptoms often result in cognitive, emotional, academic, occupational, and social impairment [4]. The disorder is notably heterogeneous since its clinical presentation can vary markedly between individuals and across the lifespan. While hyperactivity symptoms tend to diminish with age, attentional difficulties often persist during adolescence and adulthood [94,95,96,97].

Several studies have implicated BDNF in the pathophysiology of ADHD and response to stimulant treatment. However, the mechanisms through which BDNF contributes to this condition remain unclear, particularly whether alterations in BDNF levels in individuals with ADHD influence their activity. According to the existing literature, children with ADHD may show increased, decreased, or comparable BDNF levels when compared with typically developing children, or before and after treatment [10,39,91,98,99,100,101,102,103,104,105,106,107,108,109].

A recent systematic review and meta-analysis conducted by Silva de Lucca et al. (2023) [110] found no significant difference in peripheral BDNF levels between pre- and post-treatment with methylphenidate in children with ADHD, or between children with ADHD and control groups. In contrast, a previous meta-analysis by Zhang et al. (2018) [111] reported a gender difference in BDNF levels, with higher levels in males than females with ADHD. Methodological differences, in particular those relative to the analysis of BDNF levels in plasma, may contribute to these controversial results [110]. In fact, although BDNF can cross the blood–brain barrier and be detected in peripheral blood, once in plasma, it is stored by platelets, with only a small amount of free BDNF present in plasma [110]. Serum measurements of BDNF have shown less variability than plasma measurements [112,113].

Since BDNF is derived from the proteolytic processing of pro-BDNF, which exerts an opposite action, CNS modulation results from the balance between pro-BDNF and BDNF. A growing body of studies has shown that the pro-BDNF/BDNF ratio may be linked to concomitant neuronal activity [114]. The potential impact of pro-BDNF, BDNF, and their ratio on cognitive functioning and the severity of symptoms in children with ADHD, in relation to anomalies in the electroencephalogram (EEG), which are common in this population [115,116,117], was recently evaluated in a study conducted by M. M. El-Saied et al. (2023) [118]. According to the results of this study, the pro-BDNF/BDNF ratio was higher in children with ADHD, EEG anomalies, and significant cognitive and functional impairments, due to reduced BDNF levels, with no discernible variation in pro-BDNF levels, except in those with a history of febrile seizures [118]. BDNF concentrations are highest in three brain regions (hippocampus, frontal cortex, and amygdala), which are involved in attention and cognition [119]. A reduction in BDNF levels, due to a decline in the intracellular conversion of pro-BDNF, has been associated with disturbances in episodic memory in animals [120] and impairments in working memory and cognitive function in epileptic patients [121].

## 7. Neurotrophins in ASD

ASD is a heterogeneous and heritable neurodevelopmental condition, with an estimated prevalence of 1 in 132 individuals worldwide [122]. According to the DSM-5, diagnosis is based on persistent deficits in social communication and interaction, along with restricted, repetitive patterns of behavior, interests, or activities [4]. Symptom severity varies widely between individuals.

Alterations in BDNF expression may contribute to atypical brain development observed in ASD, including early brain overgrowth [123], atypical neural connectivity [124], disrupted cortical development [125], and heightened neuroinflammatory responses [126]. Moreover, BDNF dysregulation in experimental animal models has been shown to influence social interaction and anxiety [127,128]. Although numerous studies have explored peripheral BDNF concentrations as potential biomarkers for ASD, the evidence remains conflicting and lacks consistency due to differences in analytical methods, sample processing, and clinical characteristics such as age, presence of intellectual disability (ID), or autism subtype [129,130,131,132,133].

In addition, some studies have reported an altered ratio between BDNF and pro-BDNF, with the latter, when increased, impairing synaptic function and neuroplasticity, implicated in ASD symptoms [133,134].

Moreover, single nucleotide polymorphisms (SNPs) in the BDNF gene in conjunction with exposure to oxidative stress during early developmental stages have been associated with a higher risk of ASD, phenotypic variability, and differential treatment response [135,136,137,138].

## 8. Neurotrophins in Non-Syndromic Intellectual Disability

Non-syndromic intellectual disability (NSID) is primarily characterized by intellectual disability in the absence of distinctive syndromic characteristics. ID is defined by an intelligence quotient (IQ) of 70 or below and impairments in adaptive functioning, with onset before the age of 18 years, and has an overall prevalence of 1–3% across populations [139].

The role of BDNF and other neurotrophic factors in NSID remains incompletely understood, although the majority of NSID cases are thought to involve alterations in neuronal plasticity and network connectivity [140,141,142,143].

Early studies yielded inconsistent findings, likely due to methodological heterogeneity in sample size, participant age, diagnostic criteria, and matching protocols [119,125,144,145]. One more recent research, however, has reported a positive association between elevated peripheral BDNF levels and improved cognitive outcomes, including better verbal and total intelligence quotient (IQ) scores [146]. In line with this, Shaw et al. (2006) [147] observed that more intelligent children exhibited increased neuroplasticity and greater cortical thickness in late childhood—features that may be mediated by higher BDNF levels. These findings suggest that elevated BDNF expressions could support enhanced cognitive performance during key developmental windows.

Collectively, these results support the hypothesis that BDNF is a key modulator of neuroplasticity and contributes to the maintenance of neuronal networks underlying cognitive functioning. This aligns with broader neurobiological models in which BDNF is involved in long-term potentiation (LTP)-related learning during childhood and adolescence [148,149], as well as hippocampal-dependent memory formation and retrieval [150,151,152,153]. Furthermore, BDNF signaling has been implicated in executive functions such as rule learning, planning, and decision-making [154].

Finally, genetic studies have linked specific BDNF polymorphism to altered cognitive performance and attentional deficits, highlighting a potential genetic contribution to the pathophysiology of intellectual disability [146,155,156,157,158].

## 9. Neurotrophins in Tic Disorders

Tic disorders typically occur in early school years and are marked by brief, repetitive, and involuntary motor actions or vocal sounds. Symptoms usually end to reach their highest frequency and intensity between the ages of 9 and 11 [159,160]. In many cases, tics lessen or disappear in adulthood. Tic disorders include Tourette syndrome and chronic motor tic disorder, now considered variations in a single clinical spectrum [161].

Inflammation has been increasingly recognized as a critical contributor to the pathogenesis of tic disorders, partly by inducing changes in dopaminergic neurons of the striatum and leading to dopaminergic dysfunction [162]. Neurotrophic factors play a central role in modulating the immune system, acting as bidirectional mediators between neurons and immune cells to influence immunoreactivity [163,164]. Systemic inflammation is marked by elevated levels of proinflammatory cytokines, including interleukin-1β (IL-1β), interleukin-6 (IL-6), and tumor necrosis factor-α (TNF-α), which exert their effects primarily through the Nuclear Factor kappa-light-chain-enhancer of activated B cells (NF-κB) signaling pathway. The interaction between BDNF and its receptor TrkB has been shown to present neuroprotective effects by downregulating the expression of these inflammatory cytokines, alleviating the neuroinflammatory state observed in TS [165,166]. Notably, interventions such as rhynchophylline have been shown to significantly reduce IL-1β, IL-6, and TNF-α levels both in serum and central tissue, while also modulating dopaminergic transmission in the striatum, further supporting the role of neuroimmune mechanisms in the pathophysiology of tic disorders [166].

Although current evidence suggests a potential association between immunoreactivity, neurotrophic factors, and tic disorders, findings on specific biomarkers such NGF and GDNF remain inconclusive. A preliminary study by Karayagmurlu et al. (2018) [167] examined serum NGF and GDNF levels in a small pediatric cohort of 34 children aged 6–11 years, compared with a control group of equal size. The study found a positive correlation between NGF and GDNF levels, but no association between the serum levels of these neurotrophins and the severity of tic symptoms. Interestingly, NGF and GDNF levels were significantly higher in females with tic disorders compared with their male counterparts, possibly reflecting sex-specific stress responses and hormonal modulation [167]. These findings point to the complexity of neuroimmune interactions and suggest that peripheral levels of neurotrophic factors may reflect broader regulatory mechanisms in tic disorders.

In parallel, genetic studies have provided further support for the involvement of GDNF in the pathogenesis of tic disorders. The *GDNF* gene, located on chromosome 5p, has been repeatedly associated with TS through linkage analyses [168,169]. GDNF is critical for the survival and function of dopaminergic neurons in key regions such as the substantia nigra and ventral tegmental area, and is expressed by striatal interneurons (notably parvalbumin-positive and cholinergic cells), which are known to be reduced in TS [89,170,171]. A family-based association study identified a significant correlation between the minor allele of the intronic SNPs *rs3096140* in *GDNF* and tic disorders in two independent cohorts of European ancestry [172]. Disruption in GDNF signaling may compromise the survival of these interneurons, altering the excitatory/inhibitory balance within cortico-striatal circuits and contributing to tic expression [172]. Together, peripheral and genetic data suggest a multifaceted role of neurotrophic factors in the neurobiology of tic disorders, underscoring the need for further translational research in this area.

Key evidence on neurotrophin alterations and the associated neural mechanisms in NDDs is summarized in Table 2.

## 10. Potential Therapeutic Role of Neurotrophins and Other Neurotrophic Factors

The therapeutic potential of neurotrophins has been widely discussed in recent years, particularly in the context of neurodegenerative diseases affecting older populations, although results have so far been limited [178]. As a result, research has increasingly shifted toward indirect strategies aimed at enhancing or mimicking neurotrophin signaling.

In addition to the classical neurotrophins, other growth factors with neurotrophic properties have been implicated in the pathophysiology of NDDs. For instance, fibroblast growth factors (FGFs) contribute to neurogenesis, synaptic plasticity, and neuronal survival, and altered FGF signaling has been reported in ASD and ADHD [179,180]. Epidermal growth factor (EGF) regulates glial development and dopaminergic neuron maturation, with lower peripheral levels observed in some ASD cohorts [179]. Insulin-like growth factors (IGFs), particularly IGF-1, play key roles in neuronal differentiation, myelination, and synapse formation; reduced IGF-1 has been described in Rett syndrome, Fragile X syndrome, and subsets of ASD, prompting clinical trials of IGF-1 analogs [181,182]. Hepatocyte growth factor (HGF) is involved in axonal growth, synaptic reorganization, and the modulation of neuroinflammation, and experimental evidence suggests its dysregulation may contribute to cognitive and behavioral impairments in NDDs [183]. Although these molecules are not classified as neurotrophins, their overlapping biological functions and emerging evidence in NDD contexts highlight their potential as complementary biomarkers or therapeutic targets.

These additional neurotrophic factors—FGFs, EGF, IGF-1, and HGF—share several converging pathways with classical neurotrophins such as BDNF and NGF, including the activation of intracellular signaling cascades that support neuronal survival, plasticity, and synaptic remodeling. Given this functional overlap, strategies aimed at enhancing neurotrophin signaling, whether through the modulation of BDNF/NGF or augmentation of FGF, EGF, IGF-1, and HGF activity, may have complementary effects in neurodevelopmental disorders [184,185]. In this context, lifestyle and environmental interventions capable of upregulating BDNF expression have attracted particular attention.

BDNF expression can be modulated by various physiological stimuli, such as physical activity, menstrual cycle, light exposure, and osmotic and electrical stimuli [186], but acute and chronic stress, as well as epigenetic alterations like DNA methylation, can decrease its expression [187]. Among these, aerobic physical activity is one of the most effective and well-studied methods to stimulate endogenous BDNF production. In rodents, voluntary running rapidly increases BDNF and NGF expression in the hippocampus and cortex [188,189], while human studies have confirmed that endurance exercise elevates circulating BDNF levels [190] with improvements in memory function and neurogenesis [191].

Another promising strategy is nutritional modulation, particularly through omega-3 fatty acids (omega-3 FAs), which have been shown to influence peripheral BDNF concentrations. A recent systematic review and meta-analysis demonstrated that supplementation with omega-3 FAs—both from fish oil and plant-based sources like flaxseed—significantly increased serum BDNF levels compared with placebo, when administered at high daily doses (≥2000 mg). These findings support the prioritization of omega-3 FAs as potential adjunctive agents to promote neurotrophic signaling in clinical and developmental settings [192].

The translational relevance of neurotrophins and other neurotrophic factors in NDDs is highly context dependent. Circulating neurotrophic factors exhibit strong biological plausibility as biomarkers or therapeutic targets for NDDs, yet their standalone clinical utility remains limited due to marked heterogeneity across assays, cohorts, age groups, and the influence of variables such as medication use, physical activity, and the uncertain coupling between central and peripheral levels. Meta-analyses and cohort studies report small-to-moderate group differences for BDNF and NGF in ASD, but substantial between-study variance and overlapping values with controls preclude their use for diagnostic purposes. The direction of change for pro-BDNF and IGF-1 can vary depending on treatment exposure, age, and symptom burden, highlighting a pronounced context dependence [174,175,176]. Narrative syntheses in ASD also reveal inconsistent findings for IGF-1, FGFs, EGF, and VEGF, arguing against their adoption as universal biomarkers in the absence of standardized phenotyping and methodological harmonization [183]. At present, peripheral measurements of BDNF, NGF, IGF-1, EGF, FGF-2, and HGF are best regarded as research biomarkers, with potential roles in stratifying patients within specific syndromic NDDs—such as Rett syndrome and Phelan–McDermid syndrome—or serving as pharmacodynamic readouts rather than diagnostic tools or general severity markers in ASD, ADHD, or Tourette syndrome [175,176,183,185]. From a therapeutic perspective, IGF-1 pathway modulation shows the most advanced translational progress, particularly in Rett syndrome and Phelan–McDermid syndrome, where early-phase trials have reported improvements in selected behavioral and electrophysiological endpoints, although a broad efficacy in ASD is unproven and the long-term benefit–risk profile remains unclear. For BDNF, lifestyle-based interventions such as structured exercise appear to be reasonable adjunctive strategies with biological plausibility, whereas direct TrkB- or NGF-targeting approaches remain in preclinical or early translational phases [82,83,177,185]. Important limitations to clinical implementation include the pleiotropic nature of neurotrophic factors—being altered across a wide range of neurological and psychiatric conditions—which limits disease specificity, as well as the likelihood that effects are confined to biologically defined subgroups characterized by genetic background, age, sex, inflammation status, or medication exposure. Progress will require standardized assays, careful distinction between pro-BDNF and mature BDNF, and rigorous phenotyping. Moreover, long-term safety must be considered: chronic augmentation of pathways such as IGF-1 carries potential risks, including metabolic effects and theoretical tumorigenesis, and pediatric long-term safety data in NDDs are currently sparse, warranting cautious, trial-based application with appropriate surveillance. By contrast, strategies aimed at the physiological upregulation of BDNF, such as exercise, have favorable safety profiles, though their impact on core NDD symptoms remains to be firmly established [82,177,185].

These observations underscore that the biomarker and therapeutic potential of neurotrophic factors varies according to disorder-specific pathophysiology, patient characteristics, and available mechanistic evidence, warranting careful interpretation before clinical application.

## 11. Conclusions

Neurotrophins, particularly BDNF and NGF, are key modulators of brain development and synaptic plasticity, and their dysregulation has been implicated in several neurodevelopmental disorders, including ASD, ADHD, ID, and tic disorders. Although neurotrophin measurement is not yet part of routine clinical practice, emerging evidence suggests their potential as biomarkers of neurodevelopmental trajectories and as targets for non-pharmacological interventions.

From a clinical perspective, strategies that enhance endogenous neurotrophin activity—such as physical exercise, cognitive stimulation, and nutritional support—may complement existing treatments, especially in early developmental stages. Future research should focus on identifying reliable neurotrophin-related biomarkers, clarifying age- and sex-specific patterns, and exploring their role in response to intervention, with the goal of informing more personalized and developmentally tailored approaches to care.

## Figures and Tables

**Table 1:** Overview of major neurotrophins and their primary functions and roles in neurodevelopment.

	Primary Receptor	Key Functions	Role
**Brain-derived neurotrophic factor (BDNF) [82,83]**	TrkB	Synaptic plasticity, learning and memory, neuronal survival	High in early development; supports hippocampal and cortical maturation
**Nerve growth factor (NGF) [84,85]**	TrkA	Neuronal differentiation, survival of cholinergic neurons, nociception	Peripheral and central nervous system development
**Neurotrophin-3 (NT-3) [81,86]**	TrkC (TrkB)	Proprioceptive neuron development, oligodendrocyte maturation	Spinal cord and sensory neuron development
**Neurotrophin-4/5 (NT-4/5) [86,87]**	TrkB	Overlapping functions with BDNF, neuromuscular development	Supports synaptic maintenance during development
**Glial-derived neurotrophic factor (GDNF) [88,89]**	RET (via GFRα1 co-receptor)	Promotes survival of dopaminergic and motor neurons, kidney development	Critical for enteric nervous system and motor neuron maturation

**Table 2:** Neurotrophin alterations and neurobiological implications in neurodevelopmental disorders.

	Key Findings	Mechanistic Implications
**ADHD**	**BDNF**: Overall levels not significantly different from controls, but higher in males (linked to poorer cognition) and in females (linked to fewer attentional errors) [111,173]. Elevated pro-BDNF/BDNF ratio due to reduced BDNF, associated with cognitive deficits and EEG abnormalities [118]. **NT-3**: Elevated vs. controls, no link to symptom severity [105]. **NT-4**: Data insufficient. **NGF**: No consistent difference [105]. **GDNF**: Elevated; not correlated with symptom severity [105].	Sex-specific BDNF effects on attention/executive circuits; altered pro-BDNF/BDNF ratio may impair synaptic maturation. Elevated NT-3 may reflect adaptive neuroplasticity. GDNF changes may indicate compensatory neuroprotection.
**ASD**	**BDNF**: Meta-analyses show higher levels in children, linked to abnormal brain growth, connectivity changes, neuroinflammation. Lower pro-BDNF in some medicated cases [174,175]. **NT-3**: No significant difference [176]. **NT-4**: No significant difference [176]. **NGF**: Modestly higher in meta-analyses [176]. **GDNF**: Limited data, mechanistically relevant to dopaminergic survival.	Dysregulated BDNF/pro-BDNF balance may disrupt learning/memory circuits; NGF and GDNF may influence neuroimmune and dopaminergic pathways.
**NSID**	**BDNF**: Higher levels associated with better cognitive scores and cortical plasticity [177]. **Other neurotrophins**: data limited.	BDNF supports cortical development and long-term potentiation; disruption likely contributes to cognitive impairment.
**Tic disorders**	**BDNF**: Variably altered; may modulate neuroinflammation [177]. Data on **NT-3**, **NT-4**, **NGF**, **GDNF** limited.	BDNF modulates neuroimmune balance; GDNF critical for striatal interneurons and dopaminergic function.

Abbreviations: ADHD, attention-deficit/hyperactivity disorder; ASD, autism spectrum disorder; BDNF, brain-derived neurotrophic factor; EEG, electroencephalogram; GDNF, glial cell line-derived neurotrophic factor; NGF, nerve growth factor; NSID, non-syndromic intellectual disability; NT-3, neurotrophin-3; NT-4, neurotrophin-4.

## Abbreviations

The following abbreviations are used in this manuscript:

NDDs	Neurodevelopmental disorders
DSM-5-TR	Statistical manual of mental disorders, fifth edition, text revision
ADHD	Attention-deficit/hyperactivity disorder
ASD	Autism spectrum disorder
ID	Intellectual disability
MeSH	Medical subject headings
NGF	Nerve growth factor
BDNF	Brain-derived neurotrophic factor
NT-3	Neurotrophin-3
NT-4/5	Neurotrophin-4/5
GDNF	Glial-derived neurotrophic factor
p75NTR	p75 neurotrophin receptor
Trk receptors	Tyrosine receptor kinases
CNS	Central nervous system
Ras/MAPK/ERK	Rat sarcoma/mitogen-activated protein kinase/extracellular signal-regulated kinase
PI3K/Akt	Phosphoinositide 3-kinase/protein kinase B
PLCγ	Phospholipase C gamma
VEGF	Vascular endothelial growth factor
DRG	Dorsal root ganglia
PNS	Peripheral nervous system
EEG	Electroencephalogram
SNPs	Single nucleotide polymorphisms
NSID	Non-syndromic intellectual disability
IQ	Intelligence quotient
LTP	Long-term potentiation
IL-1β	Interleukin-1β
IL-6	Interleukin-6
TNF-α	Tumor necrosis factor-α
NF-κB	Nuclear factor kappa-light-chain-enhancer of activated B cells
Omega-3 FAs	Omega-3 fatty acids
SVZ	Subventricular zone
FGF	Fibroblast growth factor
EGF	Epidermal growth factor
IGF-1	Insulin-like growth factor
HGF	Hepatocyte growth factor
